# Non-fasting lipid profile determination in presumably healthy children: Impact on the assessment of lipid abnormalities

**DOI:** 10.1371/journal.pone.0198433

**Published:** 2018-06-21

**Authors:** Lukasz Szternel, Magdalena Krintus, Katarzyna Bergmann, Tadeusz Derezinski, Grazyna Sypniewska

**Affiliations:** 1 Department of Laboratory Medicine, Nicolaus Copernicus University, Collegium Medicum in Bydgoszcz, Poland; 2 Outpatient Clinic “Esculap” in Gniewkowo, Poland; Universita degli Studi di Milano, ITALY

## Abstract

**Objective:**

Despite the common use of non-fasting measurements for lipid profile in children it remains unclear as to the extent non-fasting conditions have on laboratory results of lipids measurements. We aimed to assess the impact of non-fasting lipid profile on the occurrence of dyslipidemia in children.

**Materials and methods:**

Basic lipid profile including: total cholesterol (TC), high-density lipoprotein cholesterol (HDL-C), low-density lipoprotein cholesterol (LDL-C) and triglycerides (TG), as well as small, dense-LDL-C (sd-LDL-C), apolipoprotein AI (ApoAI), apolipoprotein B (ApoB) and lipoprotein(a) [Lp(a)], were measured in 289 presumably healthy children aged 9–11 in both fasting and non-fasting condition. The clinical impact of non-fasting lipid profile was evaluated individually for each child with estimation of false positive (FP) and false negative (FN) results.

**Results:**

The highest percentage of FP results in non-fasting condition was observed for TG (42.3%) being significantly higher when compared to FN results (p = 0.003). In contrast, the highest percentage of FN results in a non-fasting state were shown for LDL-C (14.3%), but the difference was statistically insignificant when compared to FP results. When comparing fasting and non-fasting lipid profile a number of significant differences was shown for: TG (p<0.001), HDL-C (p = 0.002) LDL-C (p<0.001) and ApoAI (p<0.001), respectively. The occurrence of dyslipidemia, recognized on the basis of non-fasting lipids was significantly higher (p = 0.010) when compared to fasting lipid profile.

**Conclusions:**

A higher occurrence of dyslipidemia, based on the measurement of non-fasting lipids in children, is suggestive of possible disorders in lipid metabolism. However, accurate identification of dyslipidemia by assessment of non-fasting lipids requires the establishment of appropriate cut-off values for children.

## Introduction

A consensus statement on non-fasting and fasting lipid profile determination proposed by the European Atherosclerosis Society (EAS) and the European Federation of Clinical Chemistry and Laboratory Medicine (EFLM) was recently published [1].

International guidelines for children advise performing the initial screening of children with no risk factors for cardiovascular disease (CVD), between the ages of 9–11 [2]. According to these recommendations, it is necessary to measure both TC and HDL-C concentrations in the non-fasting blood sample, and then calculate the non-HDL (TC-HDL) value. If the initial screening has not revealed any abnormalities in test results, it is advised to perform a second screening between the ages of 17 and 21 [3]. The consensus statement of the EAS/ EFLM also discusses the pros and cons of non-fasting measurement of lipid profile. One of its advantages is the possibility of performing a comprehensive lipid panel in children, which is crucial for cardiovascular risk prediction. In this case, a significant simplification of the preanalytical phase in terms of lipid profile testing in children is very important [1].

To accurately assess lipid profile abnormalities in children, it is essential to follow the guidelines presented in 2011 by the National Heart, Lungs and Blood Institute (NHLBI) and approved by the American Academy of Pediatrics (AAP) [3]. They place emphasis on the importance of lipid profile screening among children as a proper tool for the early identification of dyslipidemia which might lead to the development of atherosclerotic lesions and are still reversible at this stage. It must to be stated that the guidelines concerning the first screening allow performance of the non-fasting lipid profile tests.

Inadequate cut-off values for lipid profile can pose problems in the identification of children with dyslipidemia. The consensus regarding non-fasting lipid measurements presented in 2016 by EAS/EFLM highlights the necessity for the proper preparation of lipids laboratory reports when considering both adequate cut-off values as well as additional information with consideration of when the subject last ate [1].

Lack of information on cut-off values for non-fasting lipid profile levels in children may lead to incorrect interpretation of test results and accordingly to the inappropriate implementation or withholding of medical treatment [1]. Therefore, we aimed to evaluate the impact of non-fasting lipid profile on the occurrence of dyslipidemia in children, especially with regard to obese or overweight subjects within this age group.

## Material and methods

### Characteristics of the study participants

This cross-sectional study was undertaken with 289 presumably healthy children aged 9–11. The recruitment of participants was carried out in October and November 2015. The study involved 152 girls (52.6%) and 137 boys (47.4%) aged 9–11. The clinical and biochemical characteristics of the study participants are presented in Tables 1 and 2.

**Table 1:** Baseline characteristics of study participants.

Clinical factor	All (n = 289)	Girls (n = 152)	Boys (n = 137)	^P(1)^
**Age** (years)^(**2)**^	10 (9–11)	10 (9–11)	10 (9–11)	0.56
**Weight** (kg)	36.5 (30.4–42.3)	35 (29–40)	38 (31–46)	0.002
**Height** (cm)	143 (137–148)	142 (135–147)	144 (139–149)	0.007
**Waist** (cm)	66 (60–73)	65 (58–71)	68 (61–78)	0.001
**Underweight**^(3)^ [BMI *percentiles* <5]	5.9 (17)	3.5 (10)	2.4 (7)	0.89
**Optimal weight** [BM *percentiles* ≥5 and <85]	70.2 (203)	39.8 (115)	30.4 (88)	0.17
**Overweight** [BM *percentiles* ≥85 and <95]	11.4 (33)	4.1 (12)	7.3 (21)	0.71
**Obese** [BMI *percentiles* ≥95]	12.5 (36)	5.2 (15)	7.3 (21)	0.8
**Overweight and obese** [BMI *percentiles* ≥85]	23.9 (69)	9.3 (27)	14.6 (42)	0.52

^**1.**^ p differences between girls and boys.

^**2**.^ Age, weight, height and waist circumference are presented as median (25–75 percentiles). ^**3**.^BMI category (according to currently accepted cut-off values) are presented as percentages and absolute values (in brackets) [2,6].

**BMI**: body mass index

**Table 2:** Biochemical characteristics of study participants.

Parameters^(^^1^^)^	All (n = 289)			Girls (n = 152)			Boys (n = 137)		
	Fasting	Non-fasting	P^(^^2^^)^	Fasting	Non-fasting	P^(^^2^^)^	Fasting	Non-fasting	P^(^^2^^)^
**TC** (mg/dL) (mmol/L)^(^^3^^)^	170.0 (149.6–188.2) 4.40 (3.87–4.87)	170.3 (151–187) 4.41 (3.91–4.84)	0.98	172.1 (152.8–187.3) 4.46 (3.96–4.85)	170.5 (153.7–187.3) 4.42 (3.98–4.85)	0.88	167.2 (147–189.9) 4.33 (3.81–4.92)	168.8 (149.6–184.6) 4.37 (3.87–4.78)	0.87
**TG** (mg/dL) (mmo/L)	70.8 (53.5–96.2) 0.8 (0.60–1.10)	91.5 (68.8–132.7) 1.03 (0.77–1.50)	<0.001	72.9 (55–90) 0.82 (0.62–1.02)	91.5 (67.9–133.4) 1.03 (0.77–1.51)	<0.001	64.8 (48.8–97.5) 0.73 (0.55–1.10)	91.5 (69.7–132.1) 1.03 (0.79–1.50)	<0.001
**HDL-C** (mg/dL) (mmol/L)	58.4 (50.8–66.8) 1.51 (1.32–1.73)	57.1 (48.9–64.9) 1.48 (1.27–1.68)	0.002	59.1 (51.2–65.9) 1.53 (1.33–1.71)	56.8 (48.6–64.3) 1.47 (1.26–1.67)	0.009	56.5 (50.2–67.8) 1.46 (1.30–1.76)	57.6 (49.3–65.4) 1.49 (1.28–1.69)	0.058
**LDL-C** (mg/dL) (mmol/L)	98.8 (82.9–117.7) 2.56 (2.15–3.05)	95.3 (79.7–110) 2.47 (2.06–2.85)	<0.001	103.6 (86–119.5) 2.68 (2.23–3.10)	96.0 (80.8–111) 2.49 (2.10–2.87)	<0.001	94.7 (81.3–116.8) 2.45 (2.11–3.03)	93.5 (77.4–109.6) 2.42 (2.00–2.84)	0.004
**sd-LDL-C** (mg/dL) (mmol/L)	18.3 (13.8–24.1) 0.47 (0.36–0.62)	18.4 (13.6–25.1) 0.48 (0.35–0.65)	0.32	18.7 (14.6–23.5) 0.48 (0.38–0.61)	18.9 (14.0–24.7) 0.49 (0.36–0.64)	0.37	17.3 (13.1–24.3) 0.45 (0.34–0.63)	17.3 (13.4–25.6) 0.45 (0.35–0.66	0.64
**Lp(a)** (mg/dL)	10.3 (5–19.9)	10.6 (9–19.7)	0.086	9.9 (5–16.3)	10.2 (9–15.5)	0.96	10.5 (7–26)	11.0 (9.2–27.4)	0.013
**ApoB** (g/L)	0.78 (0.65–0.92)	0.81 (0.72–0.91)	0.12	0.79 (0.65–0.92)	0.83 (0.72–0.91)	0.56	0.77 (0.64–0.89)	0.78 (0.69–0.89)	0.11
**ApoAI** (g/L)	1.30 (1.16–1.49)	1.20 (1.10–1.33)	<0.001	1.31 (1.14–1.49)	1.19 (1.10–1.31)	<0.001	1.28 (1.18–1.50)	1.24 (1.11–1.35)	<0.001
**non-HDL-C** (mg/dL) (mmol/L)	110.1 (91.9–127.3) 2.85 (2.38–3.29)	110.9 (92.9–128.4) 2.89 (2.42–3.33)	0.12	112.5 (94.2–128.1) 2.92 (2.44–3.32)	110.9 (95.2–130.4) 2.88 (2.50–3.38)	0.21	106.0 (90.2–126) 2.75 (2.34–3.26)	111.0 (90.2–126.3) 2.89 (2.34–3.28)	0.37

^**1**.^Results are presented as median (25–75 percentiles); statistical significance at p<0.05.

^**2**.^p - differences in measurements fasting vs.non-fasting state.

^**3**.^ Values in mg/dL were converted to mmol/L by multiplying by 0.0259 for **TC-**total cholesterol; **HDL-C** -high density lipoprotein cholesterol; **LDL-C**-low density lipoprotein cholesterol; **sd-LDL-C**-small, dense low density lipoprotein cholesterol; and by 0.0113 for **TG**-triglycerides.

**ApoAI**-apolipoprotein AI; **non-HDL-C**- non high density lipoprotein cholesterol [TC-(HDL-C)]; **ApoB**- apolipoprotein B; **Lp(a)**- lipoprotein (a).

The physical activity of study participants is described on the basis of physical education directives issued by the Minister of National Education for curricula in public schools, classes I-III. In this three-year period, minimum compulsory physical education is 290 hours, i.e. 3 hours per week, but in subsequent classes, IV-VI this number rises to 4 hours per week [4]. Data from the Central Statistical Office (GUS) regarding extracurricular activities revealed in a national study that the average child aged 2–14 spends about 2.4 hours in front of a TV screen and/or computer monitor; this time was slightly longer for boys than girls. On the other hand, physical activity was reported by almost 85% of children studied aged 6–14 [5].

### Inclusion and exclusion criteria for study participants

The children’s age (9–11) was the first inclusion criterion taken into consideration in our study, in accordance with the AAP and NHLBI guidelines, which recommend performing (non-fasting) lipid profile screening in children who are not in a high-risk group [2,6]. The second criterion was the necessity of taking two blood samples for analysis from each participant. For this, three conditions had to be met: one blood sample from each subject was taken in fasting state (in accordance with the National Cholesterol Education Program (NCEP), Adult Treatment Panel III (ATP III) guidelines-at least 8 hours since the subject last ate) [7,8]; the second was taken after eating: between the 2^nd^ and 4^th^ hour since their last food intake (in our study, breakfast was the established meal consumed before the blood sampling). It was essential to maintain a time interval between the two samplings which could not be longer than 14 days. The minimum time interval between the first and the second blood sampling in this study was 2 days [9]. The exclusion criterion was for exceeding the time limit for fasting (<8 hrs) and non-fasting (> 4 hrs) testing.

### Laboratory and anthropometric measurements

Venous blood samples for laboratory analyses were taken by a qualified nursing team with the use of a vacuum blood collection system (Becton Dickinson, Franklin Lakes, USA) gel separation tubes. In order to obtain serum, the material was centrifuged for 10 minutes at 3000 rpm. Serum was then aliquoted and stored at -70°C. The following lipid parameters: TC, TG, LDL-C, HDL-C were measured immediately, whereas an extended lipid profile, i.e. sd-LDL-C, ApoB, ApoAI, Lp(a) was performed on previously frozen samples.

Anthropometric measurements (weight, height and waist circumference) were performed on the same day as blood samples were taken. The values of height and weight were used in order to calculate BMI and BMI percentiles. To calculate BMI an online BMI calculator was used (based on the “OLAF” project) [10,11].

All laboratory tests were performed at the Department of Laboratory Medicine, Nicolaus Copernicus University, Collegium Medicum in Bydgoszcz on the Horiba ABX Pentra 400 analyzer (Horiba ABX, Montpellier, France). Reagents for sd-LDL-C (direct automated sdLDL-C kit) were supplied by Randox Laboratories (Crumlin, UK).

Calculation of the following values were performed: 1) Non-HDL-C = TC-(HDL-C); 2) remnant cholesterol = TC-(LDL-C)-HDL-C.

Dyslipidemia was defined by at least one abnormal level of serum lipid parameters: total cholesterol (TC) ≥ 4.4 mmol/L (≥ 170 mg/dL), TG for children aged 0–9 yrs. ≥ 0.85 mmol/L (≥ 75 mg/dL); 10–19 yrs ≥ 1.02 mmol/L (≥ 90 mg/dL), LDL-C ≥ 2.85 mmol/L (≥ 110 mg/dL) and HDL-C ≤ 1.17 mmol/L (≤ 45 mg/dL) according to currently accepted cut-off values for lipid parameters in children and adolescents (Table A S1 File) [2,6].

The study was approved by the Collegium Medicum Bioethics Committee (at the Nicolaus Copernicus University, KB 338/2015). Parental written consents forms were obtained from all participants before inclusion to the study.

### Statistical analysis

The number of patients in our study allows us to estimate a test power of 0.89 for fasting and postprandial lipid analysis. Therefore, we can assume that there are no statistical differences in fasting and postprandial parameters due to an actual interrelationships and is not resultant from inadequate sample size. Agreement between the distribution of investigated variables and normal distribution was evaluated by means of the W Shapiro-Wilk’s test. Parameters with normal distribution were presented as the mean ± standard deviation (SD), whereas, parameters with non-normal distribution were presented as medians and interquartile ranges (IQR). The comparison of values in the two independent groups was performed by means of the Student’s t-test (normal distribution) or the Mann–Whitney’s U test (for non-normal distribution). In order to compare the values of two related variables the Wilcoxon signed-rank test was used. Multivariate regression analysis was performed for lipid parameters (fasting and non-fasting) after adjustment for gender and anthropometric parameters. The clinical usefulness of lipid profile was assessed both fasting and non-fasting on the basis of percentage calculation of false positive or false negative test results. In all analyses, the p-value< 0.05 was considered statistically significant. Statistical analysis was performed using Statistica 12.0 PL (StatSoft Inc., Tulsa, USA) and PQStat (PQStat 1.6.2, Poznan, Poland).

## Results

Baseline characteristics of the study participants are presented in Table 1, while detailed biochemical characteristics are shown in Table 2.

Significant differences between girls and boys were noted with regard to weight, height and waist circumference. Differences in the concentration of lipid parameters between boys and girls, both in the fasting and non-fasting state were non-significant. Following BMI percentile categories, there was no statistical significance in the occurrence of being overweight and obese between girls and boys. However, the percentage of those overweight and obese in boys was higher when compared to girls (14.6 *vs* 9.3%, respectively). Table 2 presents a comparison of fasting and non-fasting lipid profile parameters in the whole study group of 289 children with comparisons in subgroups by gender. Amongst 137 boys statistically significant differences between fasting and non-fasting states were noted for TG, LDL-C, Lp(a) and ApoAI. In this group no statistical significance was observed for TC, HDL-C, sd-LDL and ApoB. These parameters which were considered statistically significant for the whole group, were analogously noted as statistically significant in girls. Significant differences for lipid parameters concentration in fasting and non-fasting state were noted for: TG (p<0.001), HDL-C (p = 0.002), LDL-C (p<0.001) and apoAI (p<0.001), whereas TC, sd-LDL-C and Lp(a) did not differ significantly in either fasting or non-fasting state, in the group as a whole.

Table 3 presents the comparison of median lipids and apolipoproteins concentrations in fasting and non-fasting state among children with a normal body mass and those who are overweight and obese, assessed on the basis of BMI percentiles.

**Table 3:** Comparison of lipid parameters in fasting vs. non-fasting state (based on BMI percentiles).

Lipid parameters^(^^1^^)^	Optimal weight (BMI ≥5<85) [N = 203]		Trend change ↑/↓	p^(^^2^^)^	Overweight and obese (BMI≥85) [N = 69]		Trend change ↑/↓	p^(^^2^^)^
	Fasting	Non-fasting			Fasting	Non-fasting		
**TC** (mg/dL) (mmol/L)	167.0 (147.3–186.7) 4.33 (3.82–4.84)	168.4 (150.3–186.3) 4.36 (3.89–4.83)	**(-)**^(^^3^^)^	0.91	175.6 (157.5–193.6) 4.55 (4.08–5.01)	174.7 (156.9–192.2) 4.52 (4.06–4.98)	**(-)**	0.92
**TG** (mg/dL) (mmol/L)	64.6 (49.4–83.9) 0.73 (0.56–0.95)	85.3 (64.6–115.0) 0.96 (0.73–1.30)	**↑**	<0.001	100.3 (75.8–130.9) 1.13 (0.86–1.48)	139.5 (97.3–179.3) 1.58 (1.10–2.03)	**↑**	<0.001
**LDL-C** (mg/dL) (mmol/L)	95.3 (81.2–116.0) 2.47 (2.10–3.00)	93.6 (77.8–106.8) 2.42 (2.02–2.77)	**↓**	<0.001	107.7 (87.6–128.7) 2.79 (2.27–3.33)	106.5 (88.8–122.1) 2.76 (2.30–3.16)	**(-)**	0.13
**HDL-C** (mg/dL) (mmol/l)	59.2 (52.1–68.6) 1.53 (1.35–1.78)	58.2 (51.3–65.7) 1.51 (1.33–1.70)	**↓**	0.002	51.5 (45.8–60.2) 1.33 (1.19–1.56)	51.7 (43.9–58.2) 1.34 (1.14–1.51)	**↑**	0.05
**sd-LDL-C** (mg/dL) (mmol/L)	17.4 (13.8–23.4) 0.45 (0.36–0.61)	17.7 (13.3–23.1) 0.46 (0.34–0.60)	**(-)**	0.10	20.5 (14.5–27.1) 0.53 (0.38–0.70)	20.6 (16.1–30.7) 0.53 (0.42–0.80)	**(-)**	0.48
**Lp(a)** (mg/dL)	10.0 (5.0–18.8)	10.2 (6.0–16.2)	**(-)**	0.63	10.9 (9.0–24.7)	11,6 (9.3–25.2)	**↑**	0.02
**ApoB** (g/L)	0.75 (0.64–0.88.)	0.78 (0.70–0.89)	**↑**	0.006	0.91 (0.75–1.06)	0.88 (0.77–0.96)	**(-)**	0.10
**ApoAI** (g/L)	1.30 (1.17–1.47)	1.22 (1.12–1.37)	**↓**	<0.001	1.28 (1.16–1.58)	1.14 (1.04–1.28)	**↓**	<0.001
**Non-HDL-C** (mg/dL) (mmo/L)	105.3 (89.1–124.0) 2.73 (2.31–3.21)	109.2 (90.6–124.6) 2.83 (2.35–3.23)	**(-)**	0.5	122.2 (105.5–137.7) 3.16 (2.73–3.57)	123.9 (106.4–139.0) 3.21 (2.76–3.60)	**(-)**	0.85

^**1**.^Results are presented as median (25–75 percentiles).

^**2**.^p significance of differences in measurements fasting vs.non-fasting state.

^**3**.^Lack of significant trend.

**BMI**-Body Mass Index; **TC**-total cholesterol; **TG**-triglycerides; **LDL-C**-low density lipoprotein cholesterol; **HDL-C**-high density lipoprotein cholesterol; **sd-LDL-C**-small, dense low density lipoprotein cholesterol; **Lp(a)-** lipoprotein (a); **ApoB**- apolipoprotein B; **ApoAI**-apolipoprotein AI; **non-HDL-C**- non high density lipoprotein cholesterol [TC-(HDL-C)].

In contrast to the group as a whole and children with a normal BMI (where the concentration of TC was slightly higher in non-fasting state), in overweight and obese children TC concentrations were slightly lower (by 0.5% fasting *vs*. non-fasting). A similar tendency for lower TC concentrations (by 0.9%) in non-fasting state was observed in girls. Significantly higher concentrations of TG in non-fasting state were found among children with a BMI≥85 (of 28.1%) compared to those with optimal BMI (24.3%). In overweight and obese children, a slight but non-significant elevation of HDL-C and a lower concentration of apoB was observed.

**Fig 1:**
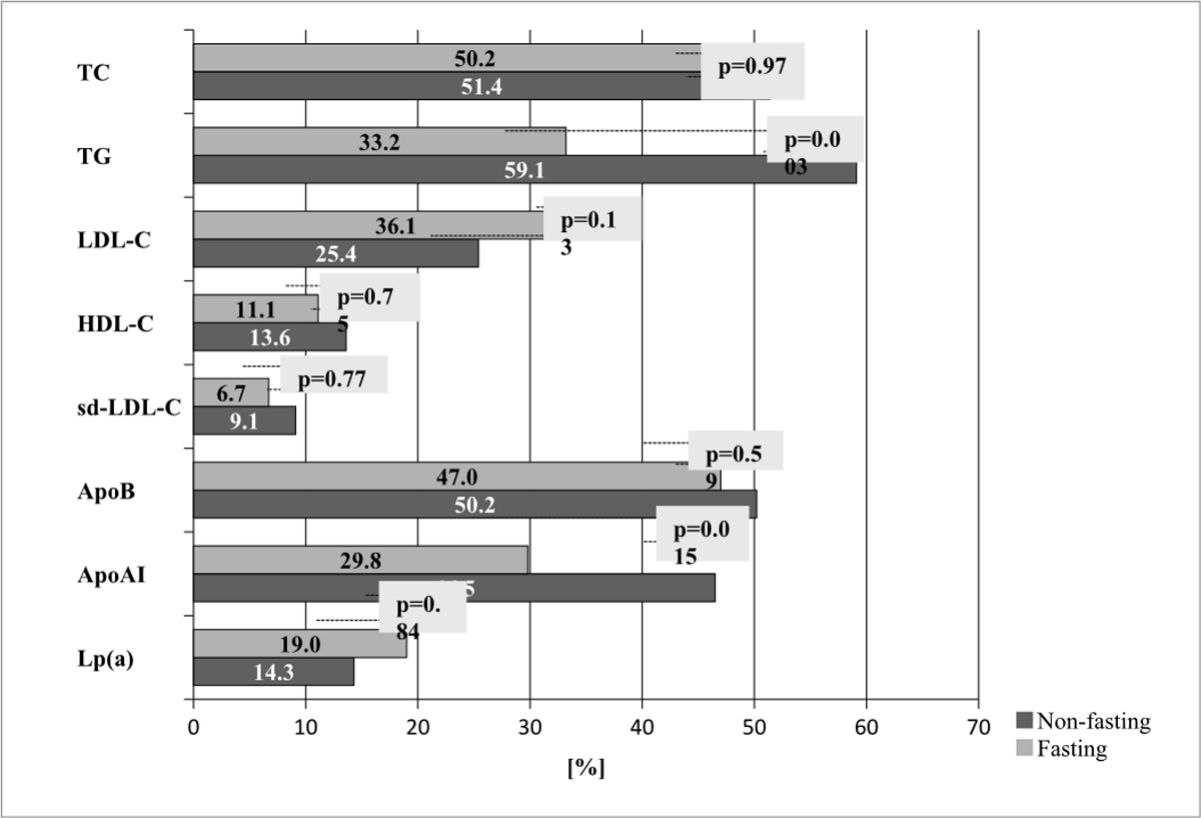
The percentage of abnormal lipid parameters results in fasting vs. non-fasting state [N = 289]. *Cut-off point for lipids in accordance with The National Heart, Lung and Blood Institute (NHLBI) guideline [2,6]. **TC**-total cholesterol; **TG**-triglycerides; **LDL-C**-low density lipoprotein cholesterol; **HDL-C**-high density lipoprotein cholesterol; **sd-LDL**-small, dense low density lipoprotein cholesterol; **Apo B**- apolipoprotein B; **ApoAI**-apolipoprotein A1; **Lp(a)**- lipoprotein (a).

The occurrence of abnormal concentrations of lipids measured in fasting *vs*. non-fasting state in children in relation to the currently recommended values for lipids and apolipoproteins in fasting condition is presented in Fig 1.

Statistically significant differences in both fasting and non-fasting states were only observed for TG (33.2% *vs* 59.1%) and ApoAI (47.0% *vs* 50.2%). In the remaining lipid parameters there were no statistically significant differences identified in the percentages of lipid abnormalities.

**Fig 2:**
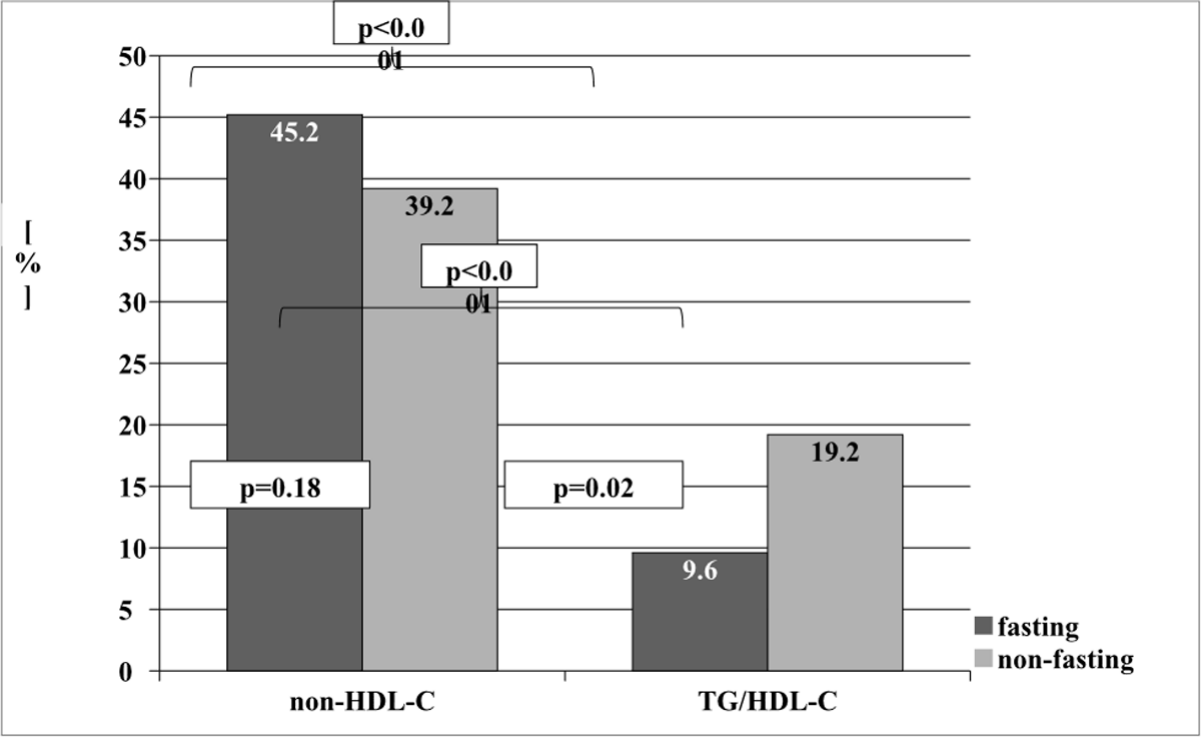
The percentage of diagnosis of dyslipidemias with the use of two lipid indices: Non-HDL-C and TG/HDL-C in fasting/non-fasting state. non-HDL-C- non high density lipoprotein cholesterol [TC-(HDL-C)]; **TG/HDL-C**–triglycerides to high density lipoprotein cholesterol ratio.

The identification of dyslipidemias based on two lipid indices was greater for non-HDL-C when compared to the TG/HDL-C ratio, in both fasting (45.2 vs 9.6) and non-fasting (39.2 vs 19.2) states (Fig 2).

In employing non-HDL-C for the recognition of lipids abnormalities, we obtained similar percentages in both fasting and non-fasting states. Though there were no significant differences regarding identification of dyslipidemia for non-HDL-C, when using Pearson's analysis, we demonstrated a stronger correlation between most of the lipids measured in fasting and non-fasting states (Table B S1 File).

When comparing the differences between percentages values for particular lipid parameters exceeding their cut-off values, we noticed that the highest percentage of abnormal lipid and apolipoproteins concentrations occurs in LDL-C (with 10.7% more abnormal results in children in fasting state) and in TG and apoAI (respectively 25.9% and 16.7% more abnormal results in children in non-fasting state). Similar changes were observed after categorization according to BMI percentiles (Table 4).

**Table 4:** Occurrence of abnormal lipid parameters: Comparison on the basis of BMI and fasting/non-fasting status.

Lipid parameters	Optimal body mass [N = 203]		p	Overweight and obese [N = 69]		p
	Fasting [%/n]	Non-fasting [%/n]		Fasting [%/n]	Non-fasting [%/n]	
**TC**	45.8 (93)	47.8 (97)	0.79	60.9 (42)	59.4 (41)	0.89
**TG**	23.6 (48)	51.2 (104)	0.001	63.8 (44)	84.1 (58)	0.01
**LDL-C**	33.0 (67)	21.2 (43)	0.18	46.4 (32)	40.6 (28)	0.65
**HDL-C**	8.4 (17)	9.4 (19)	0.92	21.7 (15)	27.5 (19)	0.70
**sd-LDL**	5.4 (11)	6.4 (13)	0.92	11.6 (8)	18.8 (13)	0.66
**ApoB**	40.0 (81)	44.3 (90)	0.56	69.6 (48)	69.1 (47)	0.88
**ApoAI**	31.0 (63)	47.8 (97)	0.04	33.3 (23)	62.3 (43)	0.02
**Lp(a)**	17.7 (36)	17.7 (36)	1.00	20.3 (14)	17.4 (12)	0.85
**non-HDL**	5.4 (11)	8.4 (17)	0.77	17.4 (12)	18.8 (13)	0.93

**BMI**-Body Mass Index; **TC**-total cholesterol; **TG**-triglycerides; **LDL-C**-low density lipoprotein cholesterol; **HDL-C**-high density lipoprotein cholesterol; **sd-LDL-C**-small, dense low density lipoprotein cholesterol; **ApoB**-apolipoprotein B; **ApoAI**-apolipoprotein AI; **Lp(a)**- lipoprotein (a); **non-HDL-C**- non high density lipoprotein cholesterol [TC-(HDL-C)].

Only TG and ApoAI showed significant differences between fasting and non-fasting states when compared to groups with optimal body mass and with BMI≥85. Interestingly, sd-LDL-C was characterized by the highest change in the overweight/obese group in fasting *vs*. non-fasting state (7.3% higher), although statistically non important.

**Fig 3:**
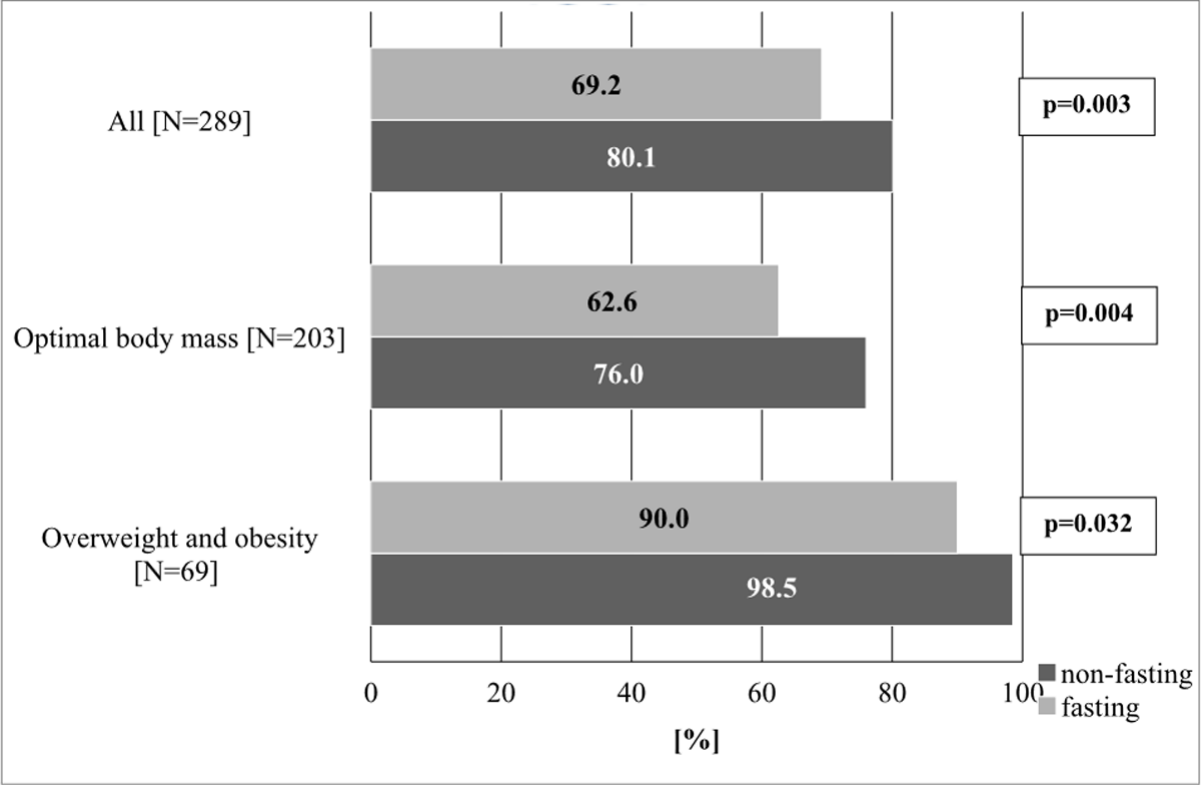
The occurrence of dyslipidemia according to BMI category. **BMI**: body mass index.

A higher occurrence of dyslipidemias, recognized on the basis of abnormal concentrations of TC, TG, LDL-C or HDL-C in a non-fasting state, was identified in both overweight/obese children and those with optimal BMI, although the differences in the former group were non-significant. The occurrence of dyslipidemia recognized on the basis of non-fasting lipids (80.1%) was significantly higher (p = 0.01) than those based on fasting values (69.2%) (Fig 3).

Multiple regression analysis revealed that non-fasting TG concentration was significantly affected by sd-LDL-C concentrations in a non-fasting state (β = 3.58, p<0.01). This model was statistically significant and explained 60% of TG variability.

**Fig 4:**
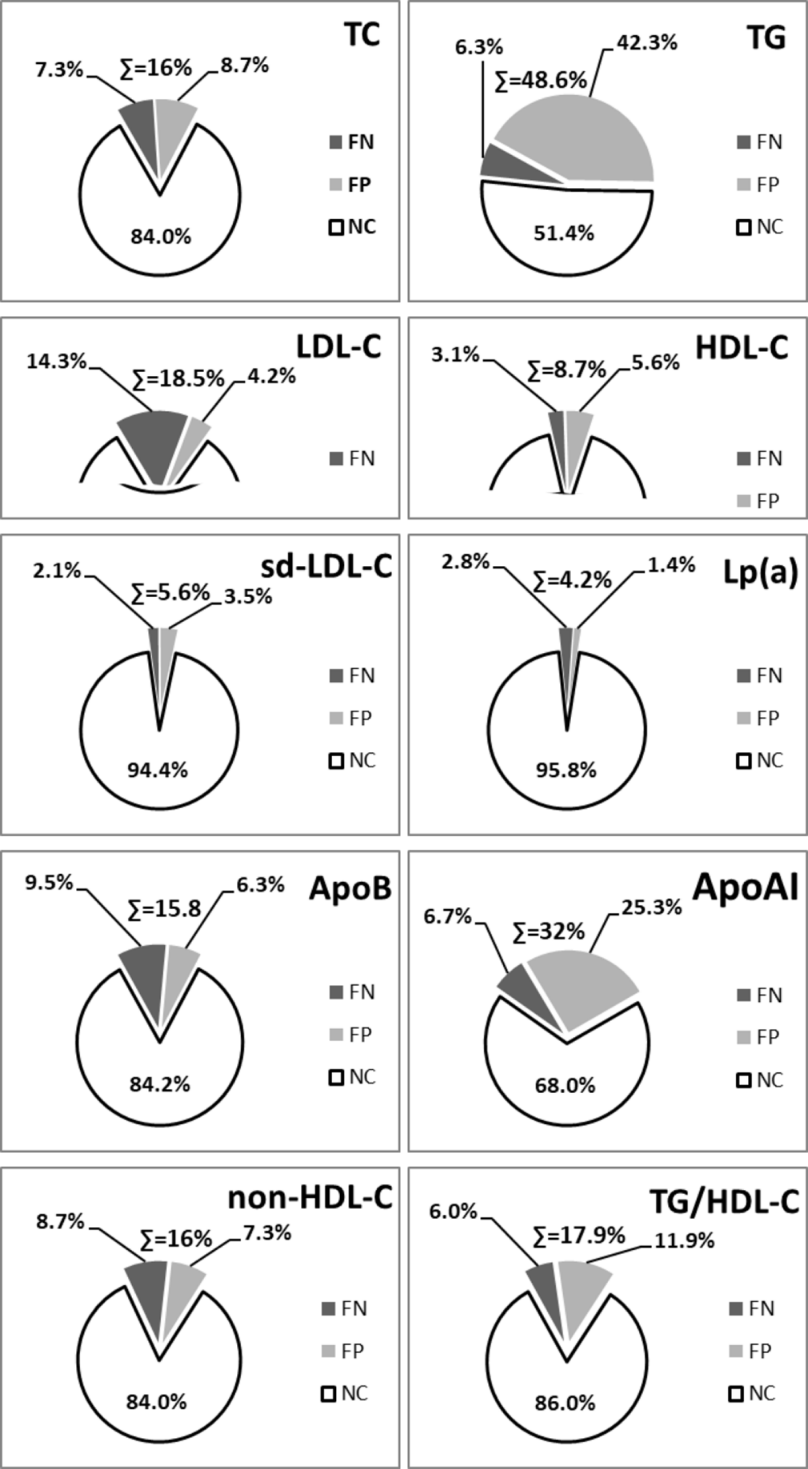
The impact of non-fasting measurements on clinical significance. **NC** (No Change)- measurements in fasting and non-fasting state showed the same lipid classification; **FP** (False Positive);**FN** (False Negative);**∑** (the sum of **FP** and **FN** results); **TC**-total cholesterol; **TG**-triglycerides; **LDL-C**-low density lipoprotein cholesterol; **HDL-C**-high density lipoprotein cholesterol; **sd-LDL-C**-small, dense low density lipoprotein cholesterol; **ApoB**- apolipoprotein B; **ApoAI**-apolipoprotein AI; **Lp(a)**- lipoprotein (a); **non-HDL-C**- non high density lipoprotein cholesterol [TC-(HDL-C)]; **TG/HDL-C**–triglycerides to high density lipoprotein cholesterol ratio.

The clinical usefulness of the results of lipid parameters measured in a non-fasting state in comparison to fasting concentrations is presented in Fig 4.

In order to individually analyze the results of lipid profiles each of the study participants was assessed in parallel, both in fasting and non-fasting states. The concentration of each measured lipid parameter in both fasting and non-fasting state was evaluated and described as normal, elevated or decreased, applying the accepted criteria. Analysis was based on the identification of false-positive (FP) results if non-fasting values were elevated relative to the fasting value, and false-negative (FN) results if non-fasting values were decreased relative to the fasting value. The largest correspondence for fasting and non-fasting measurements was noted for Lp(a) (*NC* = 95.8%), whereas the largest percentage of non-comparable results was identified for TG (*∑* = 48.6%). In the analysis of the standard lipid profile, i.e. TC, TG, LDL-C and HDL-C, the NC (No Change) between fasting and non-fasting measurements was identified as: 84%; 51.4%; 81.5% and 91.3%, respectively. The highest figures of FP and FN results was noted for TG (48.6%), whereas the lowest was for Lp(a) (4.2%). When comparing two calculated lipid indices such as non-HDL-C and TG/HDL-C we obtained closely comparable percentages for FP and FN results (16% vs 18%). The TG/HDL-C ratio was characterized by a better correlation only with ApoAI in a non-fasting state. In non-fasting children dyslipidemias were recognized almost twice as often when using non-HDL-C (39.2%) compared to TG/HDL-C (19.2%).

Youden's analysis showed that the area under the ROC curve (AUC) was significantly greater in non-fasting condition compared to fasting for: TG (AUC 0.887 vs 0.778), ApoB (AUC 0.824 vs 0.785) and TG/HDL-C ratio (AUC: 0.855 vs 0.723). Remaining lipid parameters had significantly greater AUC in fasting state, though LDL-C had no statistical difference in AUC in fasting and non-fasting conditions. Our study showed that among all analyzed lipid parameters and indices AUC were highest for non-HDL-C (AUC = 0.907) in fasting condition, although in non-fasting state non-HDL-C and TG had an equal diagnostic power as AUC = 0.887 (Table C S1 File).

## Discussion

The current NHLBI and AAP guidelines recommend that the first screening for lipid profile should be carried out in children aged 9–11, with no necessity for fasting prior to testing, with the proviso that under this conditions it is advisable to measure TC concentrations and non-HDL-C [2,12]. The question which arises when we consider measuring non-fasting lipid parameters, is how much does the non-fasting state actually influence lipid metabolism?

Our study confirmed minor, insignificant differences, between concentrations of most lipid parameters, excluding TG, LDL-C and ApoAI, measured in non- fasting versus fasting states. The study of Steiner et al, together with other similar studies, suggests that changes in non-fasting lipid profile are essentially minor and even if statistically significant, their clinical value remains negligible [13]. In our study, we aimed to verify the clinical significance of an extended lipid panel in a non-fasting state in comparison to measurements taken in fasting conditions. Due to the parallel analysis of lipids in the same children, our assessment showed a high percentage of false-positive results for non-fasting TG (42.3%), which may incorrectly imply hypertriglyceridemia. The most significant impact of false negative results measured in non-fasting conditions was observed for LDL-C (14.3%), which might result in the omission of highly atherogenic LDL-C cholesterol fraction. The relatively small impact of non-fasting state on sd-LDL-C concentration makes it an important marker of atherosclerotic processes. Our results show that sd-LDL-C is a significant marker of atherogenic triad (elevated sd-LDL-C, TG and lowered HDLC-C concentrations) in accord with Nishikura et al., who also underline the usefulness of sd-LDL particles especially in predicting and monitoring CVD [14]. The paucity of sd-LDL-C measurements in a routine lipid panel necessitates of its supplementation with an equally useful lipid marker. The strong relationship between sd-LDL-C and non-HDL-C supports the use of the latter as a surrogate for non-fasting lipid indices without incurring any additional cost [15].

The problem which is brought to the fore in implementing lipid measurements in a non-fasting state in children, is the lack of adequate cut-off values for particular lipid components. The issue of cut-offs has been widely studied but only in an adult population, resulting in the proposed EAS/EFLM reference ranges for lipids and apolipoproteins measured in a non-fasting state [1]. Khendi et al. (2015) and the EAS/ EFLM have proposed cuf-off values for lipid profile, which are optimal concentrations and are meant to be a precise tool for risk prediction of CVD whilst minimizing the danger of obtaining innacurate laboratory results [1,16,17].

In overweight and obese children there were no statistical differences in percentages for the occurrence of dyslipidemias between fasting and non-fasting states. On the other hand, in children with optimal body mass, higher occurrences of dyslipidemias were identified in non-fasting children; though this tendency was identified for all participants with optimal BMI.

In analyzing standard lipid profile which include (TC, LDL-C, HDL-C,TG) significant differences were shown for most parameters except for TC. In accord with previous studies concerning the analysis of lipid levels in a non-fasting state, our study confirmed significantly higher TG concentrations measured in non-fasting conditions (by 22.6%). We also identified a stronger association when compared to a fasting state between non-fasting TG and sd-LDL-C (β = 2.28 *vs* 3.58; p<0.01). This relationship highlighted the underestimated diagnostic value of non-fasting TG concentration as a useful tool in CVD assessment. According to Nordestgaard et al, non-fasting TG concentrations are characterized by a significant usefulness in the assessment of the risk of atherogenic changes development [18]. In addition other studies, suggest that non-fasting TG concentrations are characterized by a stronger predictive ability for CVD relative to their counterpart measured in fasting conditions [19,20]. It is noteworthy that our study highlights a higher sensitivity in the identification of hypertriglyceridemia, evidenced in our results-findings in non-fasting condition. In contrast to TG, the concentrations of LDL-C determined in a non-fasting state were significantly lower. Similarly, lower LDL-C concentrations were also observed by Steiner et al [13]. Non-fasting lower concentrations of TC were identified in girls and overweight and obese children similar to the study by Steiner et al. which confirmed lower concentrations of TC in non-fasting conditions, but without gender and BMI categorization [13]. Our study is consistent with Sidhu and Naugler’s which indicated no statistically significant changes in TC concentrations comparing fasting and non-fasting measurements [1,21]. The statistically significant differences between HDL-C concentration in a fasting and non-fasting state, were shown in the study group overall, and the subgroups of girls together with those of optimal weight. However, among the boys and children with BMI≥85, HDL-C in a non-fasting state was insignificantly higher. Lower HDL-C concentrations in a non-fasting state were identified only by Langsted et al, whereas in other studies no significant changes were observed [13,21,22,23]. Significant differences in non-fasting concentrations of extended lipid profile (which in our study include sd-LDL-C, ApoAI, ApoB, Lp [a]), were found for Lp(a) (only in boys and children with BMI≥85) and for ApoAI in the study group overall.

Our study seems to confirm the results of Varbo et al, which highlights non-HDL-C indicator as a good, independent from the fasting state marker, reflecting proatherogenic particles which are the main cause in the development of atherosclerotic changes [24]. In our study both non-HDL-C were the best predictors in the diagnosis of dyslipidemias in children in non-fasting state having an equal diagnostic power. Taking Youden’s statistics into consideration, we might assert that in our study, non-HDL-C was superior to other lipid parameters and better identified highly atherogenic particles, especially in non-fasting state. Both TG/HDL-C and non-HDL-C correlate well with the increased number of sd-LDL-C particles, however based on AUC, non-HDL-C appears to be slightly better when compared to the TG/HDL-C ratio in the diagnosis of dyslipidemias among children in non-fasting condition [15]. Yoo et al. showed TG/HDL-C to be a good predictor of cardiovascular disease in obese children and adolescents, as well as highlighting on association with insulin resistance, though recent lipid screening guidelines for children recommend non-HDL-C as a universal screening test in non-fasting condition [25]. In agreement with the Bogalusa Heart Study and the National Heart, Lung, and Blood Institute, non-HDL-C is considered a reliable marker in the screening of dyslipidemias in children [13]. The impact of non-fasting state on clinical decision making in the identification of dyslipidemias is similar for both non-HDL-C and TG/HDL-C, although non-HDL-C better reveals abnormalities in lipid metabolism, covering all atherogenic particles containing ApoB [3]. Moriyama et al. endorse the use of non-HDL-C in patients with hypertriglyceridemia (> 400 mg/dL; > 4.52 mmol/L) in the diagnosis of dyslipidemias, with strong evidence for the relationship between non-HDL-C and sd-LDL-C [26].

Existing AAP recommendations suggest the special usefulness of non-fasting non-HDL-C indicator. Among non-fasting children who are obese and overweight the only indicator which showed statistical importance was non-HDL-C. Non-HDL-C as well as remnant cholesterol assess the presence of strongly proatherogenic apolipoproteins that include ApoB particles [24,27]. Our study showed that in contrast to non-HDL-C of better diagnostic value but only in fasting conditions was observed for remnant cholesterol [28].

The indisputable utility of our study is the particular age of children involved and the nature of its parallel lipid and apolipoprotein determination in both fasting and non-fasting conditions which were not previously employed in similar comparative studies. The age range which was set between the physiological period of increasing lipid parameter concentrations before the age of 9, and the physiological decrease of lipid parameter concentrations in adolescence can be called as a “lipid diagnostic gap” [6]. The innovative nature of this study is related to individual assessment of lipid metabolism in the same individual children tested in both fasting and non-fasting states, which allowed a direct insight and comparison of the dynamics of lipid changes in non-fasting state relative to fasting conditions.

A potential limitation of our study is the lack of a standardized meal before blood sampling in the determination of lipid parameters. The only attempt at standardizing the meal was establishing its type, which in our case was breakfast. Another limitation of this study was the lack of appropriate non-fasting cut-off values for children at this age. For the purpose of this study we used cut-off values for lipids and apolipoproteins established in fasting conditions appropriate for children in this age [2,6]. Due to the lack of an adequate questionnaire specifically referring to alimentary lifestyles and physical activity we are compelled to include this field as a limitation of our study, although we would like to highlight that the parents of participating children did not receive laboratory results before the second blood collection, to avoid any bias resulting from changes to their normal activities.

## Conclusions

The higher occurrence of FP TG results in non-fasting condition as well as FN results for non-fasting LDL-C, could potentially lead to diagnostic misclassification. Due to the potentially small impact of non-fasting TC and HDL-C and their slight changes in concentration compared to a fasting state, we have underlined the usefulness of non-HDL-C in assessing proatherogenic cholesterol fractions. More frequent diagnosis of dyslipidemia among non-fasting children may be due to the lack of appropriate cut-off points. To interpret non-fasting lipid and apolipoprotein test results correctly, it is necessary to implement adequate cut-off values according to non-fasting condition, age and sex, in order to minimize false-positive or false-negative results.

## Supporting information

S1 File **Table A:** Currently accepted cut-off values for lipids in children (according to NCEP) [2,6]. **Table B:** Pearson analysis of lipid indices in non-fasting condition. **Table C:** ROC curve for lipid parameters in dyslipidemia diagnosis.
(DOCX)

## Abbreviations

TC: total cholesterol
HDL-C: high-density lipoprotein cholesterol
LDL-C: low-density lipoprotein cholesterol
TG: triglycerides
sd-LDL-C: small dense LDL-C
ApoAI: apolipoprotein AI
ApoB: apolipoprotein B
Lp(a): lipoprotein (a)
FP: false positive
FN: false negative
EAS: European Atherosclerosis Society
EFLM: European Federation of Clinical Chemistry and Laboratory Medicine
CVD: cardiovascular disease
non-HDL-C: non-HDL-cholesterol
NHLBI: National Heart, Lungs and Blood Institute
AAP: American Academy of Pediatrics
NCEP: National Cholesterol Education Program
ATP III: Adult Treatment Panel III
BMI: body mass index
SD: standard deviation
IQR: interquartile ranges
NC: no change
∑: sum of FP and FN results
