# Supplementary material for: Non-fasting lipid profile determination in presumably healthy children: Impact on the assessment of lipid abnormalities
Source: PLoS One. 2018 Jun 21;13(6):e0198433. doi: 10.1371/journal.pone.0198433 (PMC6013146; doi:10.1371/journal.pone.0198433)
Supplement: S1 File — Table A: Currently accepted cut-off values for lipids in children (according to NCEP) [2,6]. Table B: Pearson analysis of lipid indices in non-fasting condition. Table C: ROC curve for lipid parameters in dyslipidemia diagnosis. (DOCX) [file pone.0198433.s001.docx]

**Table A S1 File** Currently accepted cut-off values for lipids in children (according to NCEP) [2,6].

| **Parameters** | **Acceptable values** | |
| --- | --- | --- |
|  | **mg/dL** | **mmol/L** |
| **TC** | < 170 | <4.4 |
| **HDL-C** | > 45 | >1.17 |
| **ApoAI** | >120 | --- |
| **Non-HDL-C** | < 120 | <3.1 |
| **TG/HDL-C** | < 3 |  |
| **LDL-C** | <110 | <2.85 |
| **sd-LDL-C** | <35 | --- |
| **ApoB** | <90 | --- |
| **Lp(a)** | <30 | --- |
| **TG 0-9 *yrs.* 10-19 *yrs.*** | <75 | <0.85 |
|  | <90 | <1.02 |

**NCEP**- National Cholesterol Education Program; **TC**-total cholesterol; **HDL-C**-high density lipoprotein cholesterol; **ApoAI**-apolipoprotein AI; **non-HDL-C**- non high density lipoprotein cholesterol; **TG/HDL-C**- triglycerides to high density lipoprotein cholesterol ratio; **LDL-C**-low density lipoprotein cholesterol; **sd-LDL-C**-small, dense low density lipoprotein cholesterol; **ApoB**- apolipoprotein B; **Lp(a)**- lipoprotein (a); **TG**-triglycerides.

**-------------------------------------------------------------------------------------**

**Table B S1 File** Pearson analysis of lipid indices in non-fasting condition.

| **Lipid indicies _NF_** | **LDL-C _F_** | **LDL-C _NF_** | **sd-LDL-C _F_** | **sd-LDL-C _NF_** | **Lp(a) _F_** | **Lp(a) _NF_** | **ApoB _F_** | **ApoB _NF_** | **ApoAI _F_** | **ApoAI _NF_** |
| --- | --- | --- | --- | --- | --- | --- | --- | --- | --- | --- |
| **TG/HDL-C** | R=0.17 | R=0.17 | R=0.41 | R=0.57 | R=0.03 | R=0.05 | R=0.22 | R=-0.03 | R=-0.15 | **R=-0.38** |
|  | p=0.004 | p=0.006 | p<0.001 | p<0.001 | p=0.62 | p=0.63 | p<0.001 | p=0.63 | p=0.01 | p<0.001 |
| **Non-HDL-C** | **R=0.82** | **R=0.92** | **R=0.65** | **R=0.75** | **R=0.26** | **R=0.30** | **R=0.64** | R=-0.03 | R=0.17 | R=0.06 |
|  | p<0.001 | P<0.001 | p<0.001 | p<0.001 | p<0.001 | p<0.001 | p<0.001 | p=0.62 | p=0.005 | p=0.29 |

**F**-fasting; **NF**- non-fasting; **TG/HDL-C**- triglycerides to high density lipoprotein cholesterol ratio; **non-HDL-C**- non high density lipoprotein cholesterol [TC-(HDL-C)]; **LDL-C**-low density lipoprotein cholesterol; **sd-LDL-C**-small, dense low density lipoprotein cholesterol; **Lp(a)**- lipoprotein (a); **ApoB**- apolipoprotein B; **ApoAI**-apolipoprotein AI; **R**- Pearson correlation coefficient; **p**-statistical significance at p<0.05.

----------------------------------------------------------------------------------------------------------------------------

**Table C S1 File** ROC curve for lipid parameters in dyslipidemia diagnosis.

| **Variable** | **AUC** | **Sensitivity** | **Specificity** | **Youden index J** | **Criterion** | **P value** |
| --- | --- | --- | --- | --- | --- | --- |
| TC _F_ | 0.887 | 76.0 | 100 | 0.76 | >167.4 | 0.030 |
| TC _NF_ | 0.834 | 66.4 | 98.2 | 0.65 | >168.1 |  |
| LDL-C _F_ | 0.866 | 71.9 | 87.6 | 0.59 | >95.5 | NS |
| LDL-C _NF_ | 0.804 | 61.1 | 94.8 | 0.56 | >95.1 |  |
| non-HDL-C _F_ | 0.907 | 73.5 | 97.7 | 0.71 | >108.2 | 0.003 |
| non-HDL-C _NF_ | 0.887 | 77.3 | 91.7 | 0.69 | >103.4 |  |
| TG _F_ | 0.778 | 50.5 | 94.4 | 0.45 | >81.6 | 0.002 |
| TG _NF_ | 0.887 | 78.2 | 87.7 | 0.66 | >76.9 |  |
| sd-LDL-C _F_ | 0.800 | 72.9 | 79.3 | 0.52 | >16.9 | 0.014 |
| sd-LDL-C _NF_ | 0.789 | 59.8 | 87.9 | 0.48 | >18.4 |  |
| ApoB _F_ | 0.785 | 63.4 | 91.9 | 0.55 | >0.81 | 0.049 |
| ApoB _NF_ | 0.824 | 82.0 | 70.7 | 0.53 | >0.72 |  |
| TG/HDL-C _F_ | 0.724 | 44.0 | 96.6 | 0.41 | >1.6 | 0.001 |
| TG/HDL-C _NF_ | 0.855 | 71.6 | 96.5 | 0.68 | >1.37 |  |

**F**-fasting; **NF**- non-fasting; **NS**- non significant

**TC**-total cholesterol; **LDL-C**-low density lipoprotein cholesterol; **non-HDL-C**- non high density lipoprotein cholesterol [TC-(HDL-C)]; **TG**-triglycerides; **sd-LDL-C**-small, dense low density lipoprotein cholesterol; **ApoB**- apolipoprotein B; **TG/HDL-C**- triglycerides to high density lipoprotein cholesterol ratio.

-----------------------------------------------------------------------------------------------------------------
